# Literature information in PubChem: associations between PubChem records and scientific articles

**DOI:** 10.1186/s13321-016-0142-6

**Published:** 2016-06-10

**Authors:** Sunghwan Kim, Paul A. Thiessen, Tiejun Cheng, Bo Yu, Benjamin A. Shoemaker, Jiyao Wang, Evan E. Bolton, Yanli Wang, Stephen H. Bryant

**Affiliations:** National Center for Biotechnology Information, National Library of Medicine, National Institutes of Health, Department of Health and Human Services, 8600 Rockville Pike, Bethesda, MD 20894 USA

## Abstract

**Background:**

PubChem is an open archive consisting of a set of three primary public databases (BioAssay, Compound, and Substance). It contains information on a broad range of chemical entities, including small molecules, lipids, carbohydrates, and (chemically modified) amino acid and nucleic acid sequences (including siRNA and miRNA). Currently (as of Nov. 2015), PubChem contains more than 150 million depositor-provided chemical substance descriptions, 60 million unique chemical structures, and 225 million biological activity test results provided from over 1 million biological assay records.

**Description:**

Many PubChem records (substances, compounds, and assays) include depositor-provided cross-references to scientific articles in PubMed. Some PubChem contributors provide bioactivity data extracted from scientific articles. Literature-derived bioactivity data complement high-throughput screening (HTS) data from the concluded NIH Molecular Libraries Program and other HTS projects. Some journals provide PubChem with information on chemicals that appear in their newly published articles, enabling concurrent publication of scientific articles in journals and associated data in public databases. In addition, PubChem links records to PubMed articles indexed with the Medical Subject Heading (MeSH) controlled vocabulary thesaurus.

**Conclusion:**

Literature information, both provided by depositors and derived from MeSH annotations, can be accessed using PubChem’s web interfaces, enabling users to explore information available in literature related to PubChem records beyond typical web search results.

**Graphical Abstract:**

**Graphical abstract:**
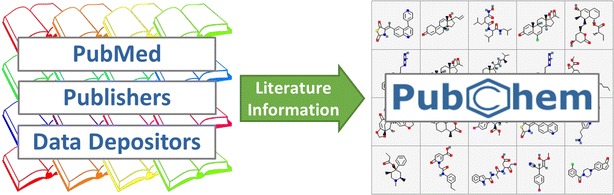
Literature information for PubChem records is derived from various sources

## Background

PubChem (https://pubchem.ncbi.nlm.nih.gov) [1–6] is an open archive which contains information on a broad range of chemical entities, including small molecules, lipids, carbohydrates, and (chemically modified) amino acid and nucleic acid sequences (including siRNA and miRNA). Since it was launched in 2004 as a component of the Molecular Libraries Program (MLP) of the U.S. National Institutes of Health (NIH), PubChem has been serving as a chemical information resource for scientific communities in many areas including chemical biology, cheminformatics, and medicinal chemistry.

Data organization in PubChem is described in detail elsewhere [6, 7], and only a brief summary is given here. Chemical information contained in PubChem is deposited by more than 350 data contributors including government agencies, academic institutions, pharmaceutical companies, chemical vendors, and publishers. PubChem organizes this information into three primary databases: Substance, Compound, and BioAssay. The Substance database (https://www.ncbi.nlm.nih.gov/pcsubstance) archives depositor-provided chemical substance descriptions. The Compound database (https://www.ncbi.nlm.nih.gov/pccompound) stores unique chemical structures extracted from the Substance database through a standardization process. The BioAssay database (https://www.ncbi.nlm.nih.gov/pcassay) contains descriptions and results of biological assay experiments. The record accessions used for the respective PubChem databases are the Substance ID (SID), Compound ID (CID) and Assay ID (AID).

As of November 2015, PubChem contains more than 150 million depositor-provided substance descriptions, 60 million unique chemical structures, and 225 million biological activity test results (from over 1 million assay experiments performed on more than 2 million small-molecules covering almost 10,000 unique protein target sequences that correspond to more than 5000 genes). It also contains RNA interference (RNAi) screening assays that target over 15,000 genes. Many of these PubChem records (substances, compounds, and assays) have depositor-provided cross-references to scientific articles in PubMed (https://www.pubmed.gov) [8–11], a biomedical literature search system developed and maintained by the National Center for Biotechnology Information (NCBI) at the National Library of Medicine (NLM), an institute within NIH.

PubMed, whose primary identifier is the PubMed ID (PMID), provides free access to more than 25 million scientific abstracts covering the fields of medicine, nursing, dentistry, veterinary medicine, health care systems, and preclinical sciences. Nearly 90 % of the PubMed contents are from MEDLINE [11, 12], which is the NLM’s bibliographic database containing more than 22 million abstracts of journal articles in life sciences with a concentration in biomedicine. A distinctive feature of MEDLINE is that the records are “indexed” with Medical Subject Headings (MeSH) [13, 14]. MeSH is the NLM’s controlled vocabulary thesaurus, consisting of sets of terms naming descriptors in a hierarchical structure. Indexing of scientific papers with MeSH terms enables users to perform a literature search at various levels of specificity. Of keen interest to PubChem is that MeSH includes a large number of chemical substance concepts, chemical names associated with each concept, and specific/qualified links between these concepts and PMIDs. Considering PubChem contains many chemical names, MeSH allows PubChem records to be linked to the biomedical literature using matching chemical names.

There are multiple sources of links to PubMed articles within PubChem and different contexts where they can be specified within a given PubChem record type. Understanding where these links can be provided and who provides them can help aid in their use. For example, they can provide PubChem users quick access to the original source article of a bioactivity result. In some cases, data contributors provide chemical information extracted from the scientific literature through manual curation or data mining. In addition, PubChem performs an automated process that annotates PubChem records with MeSH terms (by means of chemical name matching), creating associations between PubChem records and PubMed articles that share the same MeSH annotation. This work summarizes the various sources of PubMed links in PubChem, explains ways to access these links, and examines the scope of literature information associated with PubChem records.

## Construction and content

### Depositor-provided cross-references to scientific articles

#### Cross-references from substances/compounds to PubMed articles

Table 1 summarizes the number of depositor-provided cross-references (links) from PubChem substances and compounds to PubMed articles (N_SID-PMID_ and N_CID-PMID_, respectively) as well as the associated number of substances, compounds, and PubMed article records (N_SID_, N_CID_, and N_PMID_, respectively). As of November 2015, PubChem has more than five million depositor-provided cross-references (N_SID-PMID_ = 5,614,567) from 300 thousand substances (N_SID_ = 301,358) to two million PubMed articles (N_PMID_ = 2,192,601). Cross-references from compounds to PubMed articles are derived from corresponding substances. Given that compounds are the unique chemical structures extracted from the Substance database (and there can be many substance records for the same chemical), one cross-reference from a compound to a PubMed article is counted if one (or more) of the compound’s corresponding substances contains a cross-reference to that given PubMed article. Therefore, the count of compounds with PubMed cross-references is less than the count of substances with cross-references (N_CID_ = 261,497 vs. N_SID_ = 301,358). Similarly, the count of CID-PMID cross-references is less than the count of SID-PMID cross-references (N_CID-PMID_ = 5,412,256 vs. N_SID-PMID_ = 5,614,567).

**Table 1:** Summary of depositor-provided cross-references to PubMed articles from PubChem substances and compounds

	Number of cross-references		Number of records involved		
	N_SID-PMID_	N_CID-PMID_	N_PMID_	N_SID_	N_CID_
All	5,614,567	5,412,256	2,192,601	301,358	261,497
IBM Almaden Research Center^a^	5,196,617	5,125,878	2,107,354	152,777	147,576
Comparative toxicogenomics database^b^	226,585	111,029	110,000	14,463	7856
NIAID ChemDB^c^	144,477	133,012	11,951	114,953	104,418
IUPHAR/BPS guide to PHARMACOLOGY^d^	14,309	11,250	7163	6398	4913
Human metabolome database^e^	13,998	13,971	10,414	1788	1781
Immune epitope database (IEDB)^f^	4849	3863	1747	2067	1948
BioCyc^g^	3318	3267	1386	2989	2939
DrugBank^h^	3249	3225	3158	1044	1030
Biocatalysis/biodegradation database (BBD)^i^	2299	2299	644	1343	1342
Bioinformatics and drug design (BIDD) group^j^	2270	2262	91	1768	1673
Others	2596	2200	1180	1768	1448

N_SID_ and N_CID_ are the number of PubChem substances and compounds with depositor-provided cross-references to PubMed articles, respectively, and N_SID-PMID_ and N_CID-PMID_ are the number of depositor-provided cross-references from PubChem substance and compound records to PubMed articles, respectively. N_PMID_ is the number of unique PubMed articles associated with the PubChem records via the depositor-provided cross-references

^a^ http://www.research.ibm.com/labs/almaden/index.shtml

^b^Ref. [15]. http://ctdbase.org

^c^ http://chemdb.niaid.nih.gov

^d^Ref. [16]. http://www.guidetopharmacology.org

^e^Ref. [17]. http://www.hmdb.ca

^f^Ref. [18]. http://www.iedb.org

^g^Ref. [19]. http://biocyc.org

^h^Ref. [20]. http://www.drugbank.ca

^i^Ref. [21]. http://eawag-bbd.ethz.ch

^j^ http://bidd.nus.edu.sg

The biggest source of SID-PMID cross-references is the IBM Almaden Research Center (http://www.research.ibm.com/labs/almaden/index.shtml). This PubChem depositor provides PMIDs of the scientific articles mentioning a particular chemical. While beyond the scope of this paper, they also provide links to patent documents mentioning a particular chemical. The IBM substance records provide more than 90 % of the SID-PMID cross-references involving about half of all substance records containing cross-references to PubMed articles.

The next largest sources of depositor-provided literature links for PubChem Substance and Compound records are ChemDB [http://chemdb.niaid.nih.gov/—provided by the U.S. National Institute of Allergy and Infectious Diseases (NIAID)] and the Comparative Toxicogenomics Database (CTD) (http://ctdbase.org/) [15], which provide 144 thousand cross-references for 114 thousand substances and 226 thousand cross-references for 14 thousand substances, respectively. Other notable contributors for biologically relevant SID-PMID cross-references include IUPHAR/BPS Guide to Pharmacology (http://www.guidetopharmacology.org) [16], Human Metabolome Database (HMDB) (http://www.hmdb.ca/) [17], Immune Epitope Database (IEDB) (http://www.iedb.org/) [18], BioCyc (http://biocyc.org/) [19], DrugBank (http://www.drugbank.ca/) [20], and the Biocatalysis/Biodegradation database (BBD) (http://eawag-bbd.ethz.ch/) [21].

The disproportionate number of cross-references and chemical substance coverage coming from a handful of data contributors (Table 1) points to their diverging focus areas in data collection. For example, IBM uses data mining to collect information on patents and scientific papers that mention a very broad range of chemicals, resulting in a large number of links to scientific papers. On the other hand, DrugBank primarily focuses on U.S. Food and Drug Administration (FDA) drugs (approved and experimental) and collects relevant information through manual curation. Similarly, the IEDB resource performs manual curation but considers chemical substances in the immunology epitope research domain. As such, PMID cross-references provided by different chemical substance data depositors can have a significantly varied focus and approach.

#### Bioactivity data extracted from scientific articles

Data contributors may supply PubChem with a list of PMIDs for scientific articles that have information relevant to a given assay record. These articles may provide various kinds of information related to the assay, such as assay detection method, experimental protocols, assay targets, diseases associated with the targets, known ligands that bind to the targets, and so on. These articles may or may not be relevant to substances and compounds tested in those assays. With that said, it is of particular interest that some PubChem contributors provide bioactivity information extracted from the literature through the means of manual curation or data mining. These data are an important source of bioactivity information in PubChem that complements high-throughput screening (HTS) data from the now-concluded NIH Molecular Libraries Program. Table 2 shows major contributors that provide literature-extracted bioactivity data.

**Table 2:** Summary of cross-references from literature-extracted bioassay data to PubMed articles

Source	N_AID-PMID_	N_SID-PMID_	N_CID-PMID_	N_PMID_	N_AID_	N_SID_	N_CID_
ChEMBL^a^	829,503	1,068,347	1,058,637	55,582	828,594	860,191	849,149
PDBbind^b^	6946	20,221	16,993	5252	4	10,543	8158
IUPHAR/BPS guide to pharmacology^c^	442	1088	1080	151	55	273	264
BindingDB^d^	143	3114	3113	121	19	3101	3098
GLIDA^e^	–	–	–	–	6	19,474	19,458

N_AID_, N_SID_ and N_CID_ are the number of PubChem assays, substances and compounds extracted from scientific articles, respectively; and N_AID-PMID_, N_SID-PMID_ and N_CID-PMID_ are the number of cross-references from PubChem assays, substances, and compounds to PubMed articles, respectively. N_PMID_ is the number of unique PubMed articles from which the assay data are extracted

^a^Ref. [22]. https://www.ebi.ac.uk/chembl/

^b^Ref. [23]. http://www.pdbbind-cn.org

^c^Ref. [16]. http://www.guidetopharmacology.org

^d^Ref. [26]. https://www.bindingdb.org

^e^Ref. [27]. http://pharminfo.pharm.kyoto-u.ac.jp/services/glida/

ChEMBL (https://www.ebi.ac.uk/chembl/) [22] is a public database of bioactivity information for compounds. Most of the bioactivity data contained in ChEMBL is manually extracted from the full text of peer-reviewed scientific papers published in about 50 journals in the medicinal chemistry and natural product domains. From each paper, detailed information is extracted on the compounds tested, the assays performed, and the targets for the assays. Often multiple data sets are generated for a single paper, resulting in multiple BioAssay records for that publication. The ChEMBL data is updated on a regular basis, with periodic releases approximately every 3–6 months. The current version of ChEMBL (ChEMBL v20, as of September 2015) contains 13 million bioactivities for 1.5 million unique compounds, abstracted from about 60 thousand publications or provided by other data sources. ChEMBL contributes its literature-derived data to the PubChem BioAssay database.

The Guide to Pharmacology (http://www.guidetopharmacology.org) [16], created under the auspices of the International Union of Basic and Clinical Pharmacology (IUPHAR) and the British Pharmacological Society (BPS), provides in-depth integrated views of the pharmacology, genetics, functions, and pathophysiology of important drug targets, including G-protein-coupled receptors (GPCRs), ion channels, and nuclear hormone receptors (NHRs). In addition, it provides information on the interactions between these target proteins and their ligands. PubMed citations containing the bioactivity data for these ligands are also provided to PubChem.

The PDBbind database (http://www.pdbbind-cn.org) [23] collects experimentally measured binding affinity data (IC_50_, K_d_, or K_i_) for biomolecular complexes in the Protein Data Bank (PDB) [24]. The majority of the data in PDBbind is for complexes between proteins and small molecule ligands, although it also contains other types of complexes such as protein–protein, protein–nucleic acid, and nucleic acid–ligand complexes. All binding affinity data in PDBbind are manually curated from nearly 24,000 original references. PDBbind contributes to PubChem binding affinity data for ~10,000 protein–ligand complexes that involve ~3000 unique small molecule chemical structures, with related information including the PMID for the source article, PDB ID and MMDB ID [25] for the protein–ligand structures, and the protein GI and name for the protein target.

BindingDB (https://www.bindingdb.org) [26] provides measured binding affinities, focusing chiefly on the interactions of proteins considered to be drug targets with small, drug-like molecules. BindingDB contains more than one million binding interactions for seven thousand protein targets and 495 thousand small molecules.

GLIDA (GPCR-Ligand Database) (http://pharminfo.pharm.kyoto-u.ac.jp/services/glida/) [27] is a public chemical genomics database that provides information on GPCRs, their ligands, and interactions between them. The ligand-binding information was manually collected and curated from PubMed and other sources. Although GLIDA has ceased updates since 2010, it is still operational and its data are fully accessible.

In addition to these resources containing manually curated sources of bioactivity, there is a growing number of AID-PMID references deposited directly by the authors of scientific publications uploading their research data into the PubChem BioAssay database. This includes researchers from RNAi screening and is open to all researchers in the chemical biology, medicinal chemistry, and related fields. Such submissions help to satisfy open access and data sharing requirements from research funding agencies.

Note that different PubChem BioAssay data sources can provide PubMed links in different ways. For example, ChEMBL provides the cross-reference between an assay and the article from which bioactivity information for that assay were extracted, and therefore, literature evidence on bioactivity data for substances tested in that assay can be deduced from the AID-PMID cross-reference. PDBbind provides information on the source article of bioactivity data for each substance tested in an assay (as a property of the tested substance reported in the assay data table). GLIDA does not explicitly provide literature information for assays or tested substances in them. Instead, it provides the GLIDA ID (the database identifier used in GLIDA) for a substance. This external ID is used to search for the corresponding record in GLIDA to get information on the source article.

#### Chemical information provided by journal publishers

Publishers are also an indirect source of records in PubChem. While many funding agencies mandate that data from studies they support should be freely available to the public, some journals also require data submission of research data to public databases as a precondition to publishing a research paper. Data sharing requirements by journals and granting agencies may be satisfied by use of the PubChem data archiving platform.

**Fig. 1:**
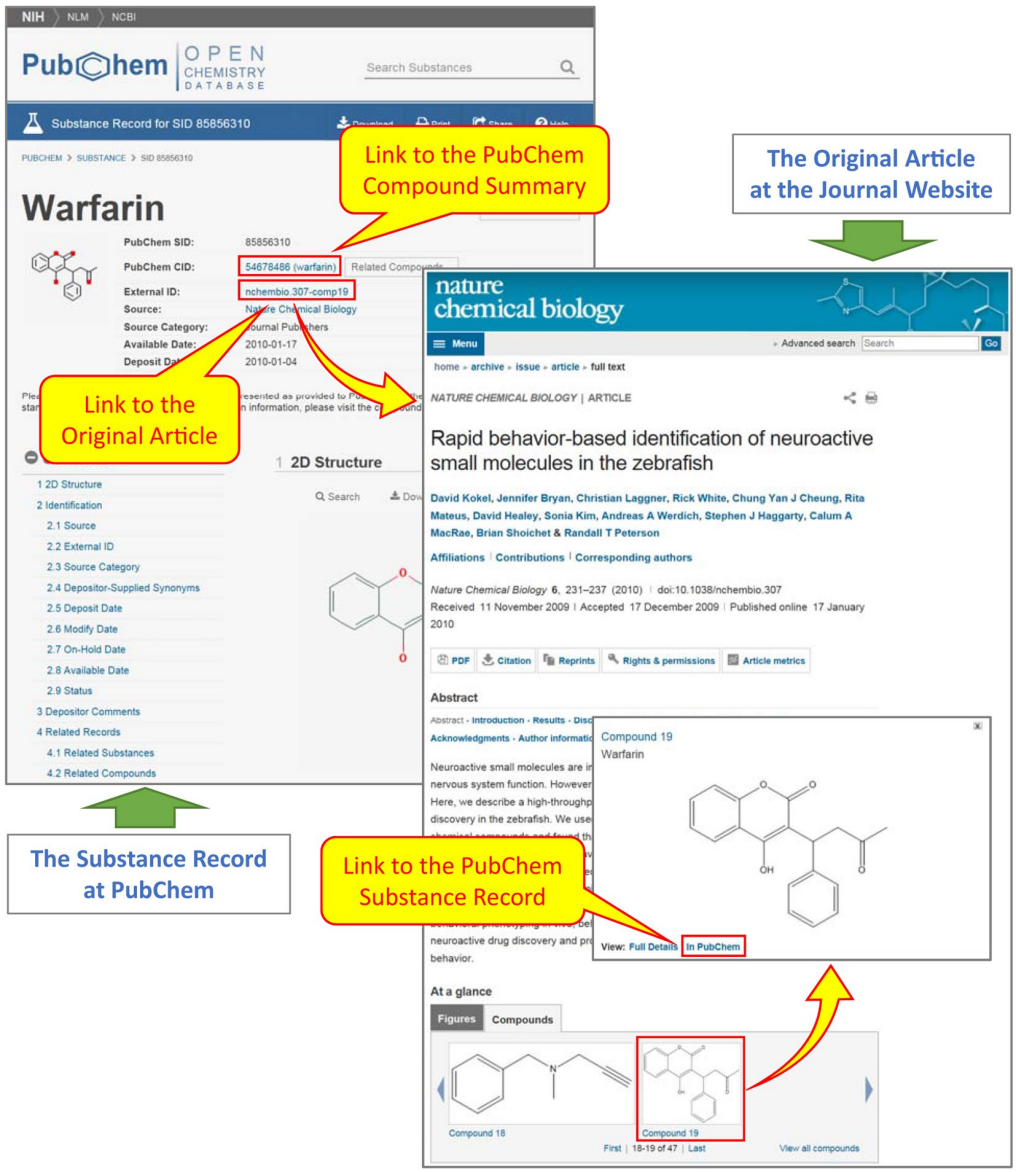
The substance record page for SID 85856310 (warfarin), with a link to the source article published in Nature Chemical Biology. The original article has a link to SID 85856310 in PubChem, allowing article readers to access comprehensive information on warfarin available in the PubChem Compound database, by clicking a link to the Compound Summary page for CID 54678486

Some journals directly provide PubChem with literature information for chemicals. Examples include journals published by the Nature Publishing Group (including Nature Chemical Biology, Nature Chemistry, and Nature Communications). When a new article is published in one of these journals, the publisher identifies chemicals studied in the article and submits them to PubChem with information on the source article (including the journal name, authors, title, publication date, and digital object identifier (DOI)). These chemicals are stored in the Substance database, and the substance record page for each chemical has a link to the source article (see Fig. 1). Similarly, on the web page of the original article the publisher provides links to the corresponding substance records in PubChem. This allows readers to readily access comprehensive information available in PubChem for these chemicals, through the compound records associated with the substance records. This demonstrates the benefit of concurrent publication of scientific articles in journals and associated data in public databases.

### Automated annotations of PubChem records with PubMed articles via MeSH

PubChem generates CID-PMID associations via MeSH using depositor-provided chemical names. This is achieved by first determining which depositor-provided chemical names in PubChem are also found in MeSH. Not all chemical names in PubChem are used in this step. Chemical names provided to PubChem are passed through a sort-of crowd-sourcing filtering process, where consistency of chemical name-structure associations of depositor-provided synonyms is determined using differing levels of chemical structure sameness (e.g., potentially grouping different charge states or salt forms of the same chemical). This reduces the count of PubChem Compound records associated with a given chemical name. The resulting filtered chemical names are considered “filtered synonyms” in PubChem.

Once PubChem chemical names are matched to MeSH concepts, the MeSH concept is associated with the PubChem Compound record containing matching chemical names. Additional consistency filtering suppresses multiple MeSH concepts from being associated with the same PubChem Compound record (by taking the consensus MeSH concept for a given compound record). This helps to reduce errors but also helps to provide a consistent chemical representation for a given MeSH chemical concept.

The CID-PMID associations are generated in a final step. PubMed records that are annotated with a given MeSH chemical concept are then associated with the PubChem Compound record linked to the MeSH chemical concept. In this way (CID→Name→MeSH→PMID), linking between PubChem and PubMed is achieved.

**Fig. 2:**
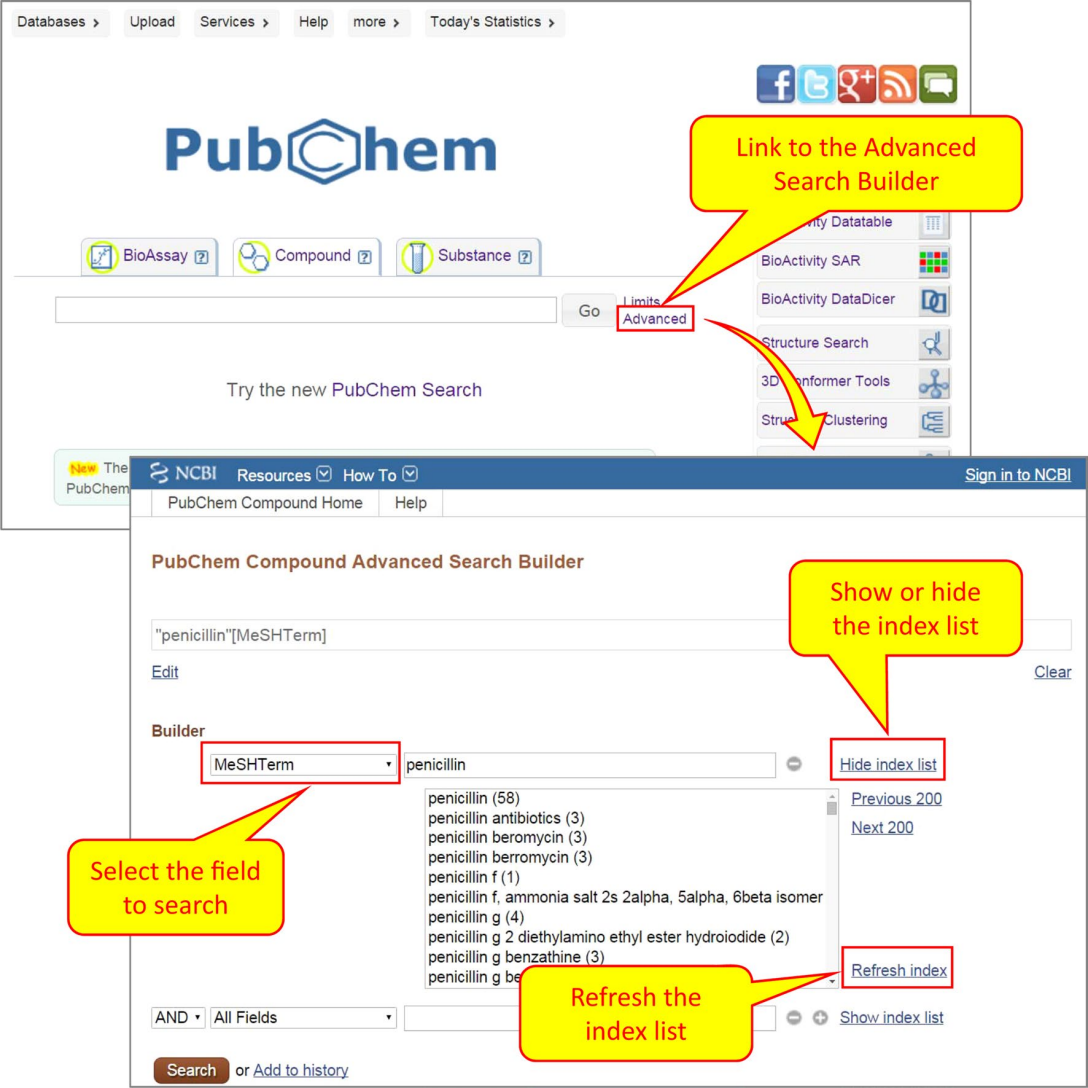
Retrieving compound records annotated with MeSH terms using the Advanced Search Builder. Clicking the “Advanced” link under the “Compound” tab on the PubChem Homepage directs users to the PubChem Compound Advanced Search Builder. Selecting the “MeSHTerm” from the dropdown menu and providing a MeSH term in the search box will retrieve compounds annotated with that MeSH term

The MeSH annotations of PubChem records are “indexed” as indices for text search within NCBI’s Entrez search system [1, 28, 29]. Entrez is the text/numeric search and retrieval system that integrates PubChem’s three primary databases (BioAssay, Compound, and Substance) with approximately forty other NCBI databases, including PubMed and MeSH. The Entrez indices are tied to individual records in PubChem and include information on particular aspects (often referred to as fields) of the records. The available fields and their indexed terms in PubChem can be explored on the Advanced Search page as part of the Search Builder (see Fig. 2).

The Entrez indices derived from MeSH annotations are summarized in Table 3. The “Complete MeSH Term” Entrez index retrieves compounds annotated with the MeSH term that exactly matches the query, while the “MeSH Term” index searches for those with the MeSH terms that partially match the query. The “MeSH Tree Node” index finds compounds annotated with the query MeSH term and those with any MeSH terms that correspond to the child nodes beneath the node for the query term. The “MeSH Description” index searches the descriptions of MeSH terms for the query string.

**Table 3:** Entrez indices used to search for records with MeSH annotations

Entrez index	Description
Compound database	
Complete MeSH term	Retrieve compounds annotated with the MeSH term that *exactly* matches the query
MESH term	Retrieve compounds annotated with MeSH terms that *partially* match the query. Note that MeSH entry terms (synonyms for the Medical Subject Heading term) are also indexed
MeSH tree node	Retrieve compounds annotated with the MeSH term that match the query and those annotated with any MeSH terms beneath the node corresponding to that MeSH term. For example, “Penicillins[MeSHTreeNode]” will retrieve records annotated with MeSH term “Penicillins”and those with MeSH terms “Oxacillin”, “Cloxacillin”, and so on, which correspond to child nodes beneath the “Penicillins” node in the MeSH tree
MeSH description	Retrieve compounds annotated with the MeSH terms whose description contains the query string
PharmAction	Retrieve compounds annotated with the Pharmacological Action term, which are a subset of MeSH terms
PharmActionID	Retrieve compounds annotated with the Pharmacological Action term corresponding to the numeric identifier given as a query
BioAssay database	
MeSH term active	Retrieve assays in which only an active substance is annotated with the MeSH term given as a query
MeSH term tested	Retrieve assays in which any tested substance is annotated with the MeSH term given as a query
MeSH description active	Retrieve assays in which only an active substance is annotated with the MeSH terms whose descriptions have a query string
MeSH description tested	Retrieve assays in which any tested substance is annotated with the MeSH terms whose descriptions have a query string
Pharm action active	Retrieve assays in which only an active substance has the Pharmacological Action annotation given as a query
Pharm action tested	Retrieve assays in which any tested substance has the Pharmacological Action annotation given as a query

Some MeSH annotations (such as solvents, carcinogens, inhibitors, and so on) are too general to describe a specific biological function of a compound. Therefore, “Pharmacological Actions” (a subset of MeSH terms) are separately included as an Entrez index, as these annotations indicate specific biological roles of chemical concepts. The “Pharmacological Action ID” index searches for records with the Pharmacological Action annotation corresponding to a numeric identifier assigned by MeSH given as a query.

MeSH annotations of a compound can be used to annotate substance records corresponding to that compound. PubChem annotates substance records with MeSH terms for Entrez searching purposes similar to compound records. However, no attempt is made to filter chemical names or MeSH concepts for substance records, using chemical name information directly provided by individual data contributors for a given substance. In addition, the MeSH annotations of substances are not displayed on the substance record page, to avoid confusion about the provenance of displayed information.

MeSH annotations to compound records made by PubChem are used to annotate assay records testing the compounds. They are also used to generate Entrez indices that allow one to search the PubChem BioAssay database for assays that have tested compounds with a particular MeSH annotation. For example, the “MeSH Term Tested” index retrieves assay records in which *any* tested compound is annotated with the query MeSH term. The “MeSH Term Active” retrieves assays in which *only* active compounds are annotated with the query MeSH term.

## Utility and discussion

### Entrez links from PubChem records to PubMed articles

The associations between PubChem records and PubMed records, whether provided by data contributors or computationally derived via common MeSH annotations, are available to PubChem users as Entrez links. In general, Entrez links are crosslinks between records in different Entrez databases or within the same Entrez database. The Entrez links provide a way to discover relevant information in Entrez databases based on a user’s specific interests. For example, one can readily obtain a list of scientific articles related to a particular molecule through an Entrez link from the corresponding compound record in PubChem to associated articles in PubMed. As another example, users may access all data sets (AID) in the BioAssay database associated with a research article in PubMed. Entrez links from PubChem to PubMed records are available to users in several ways.

#### Entrez links on the document summary (DocSum) page

**Fig. 3:**
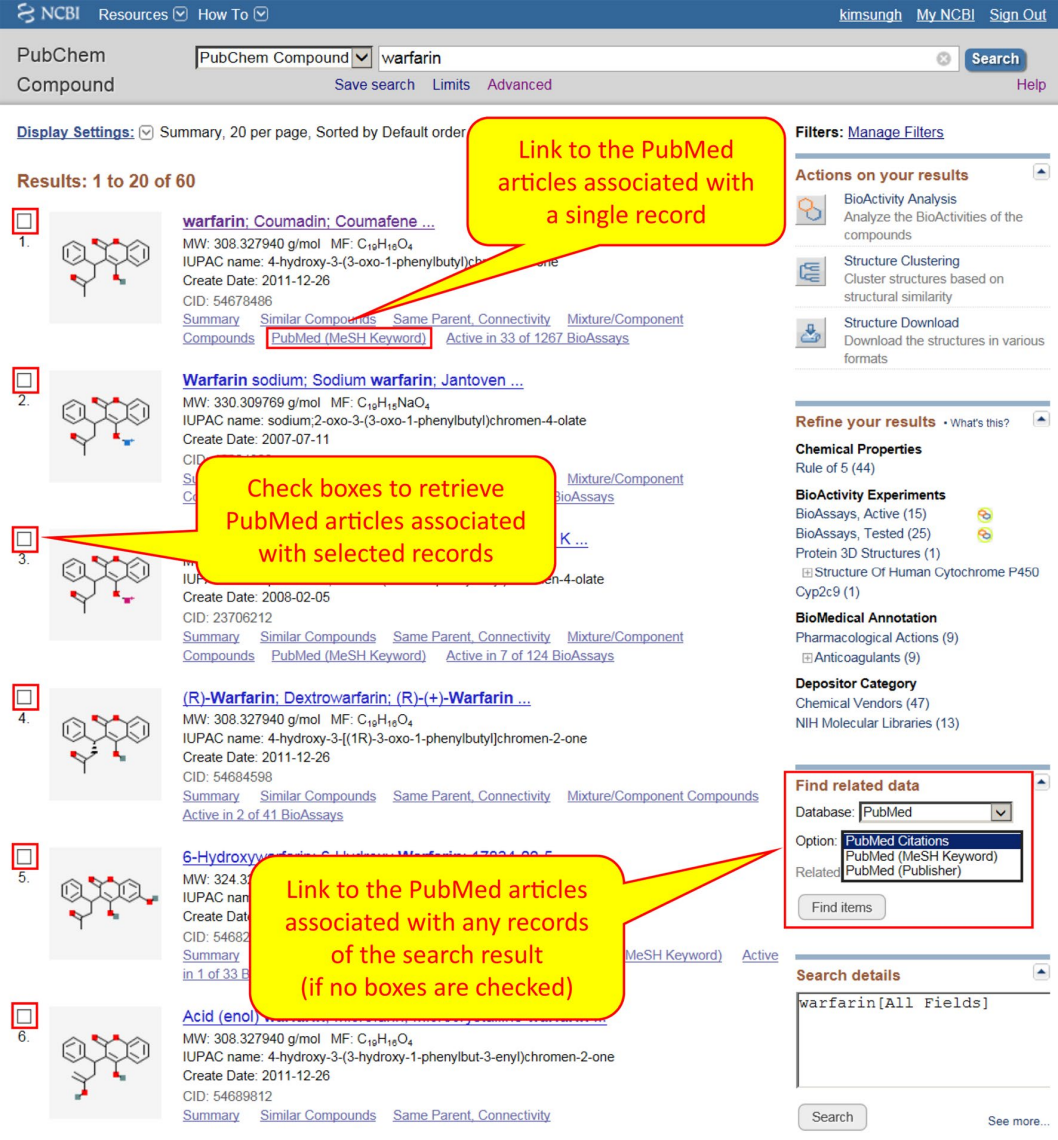
The document summary (DocSum) page that shows the results for a search for “warfarin”. Scientific articles associated with the returned compound records can be accessed via the Entrez Links, which are available under the “Find related data” menu (for multiple records) or from a link for individual compound records

An Entrez document summary (DocSum) page, as shown in Fig. 3, displays multiple records returned from an Entrez search. Entrez links are available via the “Find related data” menu on the right column. If no compounds are selected, these links will be applied to the entire search result list by default. Note that, when “PubMed” is selected from the Database drop-down menu, three items appear under the “Option” menu: “PubMed Citations”, “PubMed (MeSH Keyword)”, and “PubMed (Publisher)”. These options correspond to different types of Compound-to-PubMed Entrez Links.

The first option “PubMed Citations” is for Entrez links derived from cross-references to PubMed articles that data contributors provided to PubChem. That is, this option will retrieve scientific articles containing information relevant to a given chemical, according to PubChem depositors. On the other hand, the third option, “PubMed (Publisher)”, is from CID-PMID associations provided by journal publishers to *PubMed* (not *PubChem*) as a part of PubMed abstract submission. Both the first and third options are similar in the sense that some organization (e.g., data contributors or journal publishers) contributed the chemical-literature associations. However, the second option “PubMed (MeSH Keyword)” corresponds to CID-PMID associations derived via MeSH annotations. This option returns articles annotated by Medline indexers with MeSH terms (chemical names). MeSH annotation can be further leveraged to find which articles have a given MeSH term as a major topic to obtain articles specifically about that chemical.

Three different types of Entrez Links also exist for substance records to PubMed articles, similar to the Compound-to-PubMed links. For the BioAssay-to-PubMed links, only one type of Entrez link to PubMed is available through the right column of the DocSum page. This returns publications associated with the BioAssay records, based on AID-PMID associations provided by data contributors to PubChem. These publications may be either source articles from which assay data are collected in curation projects, or general articles that provide background information relevant to the assay.

#### Entrez links on the Compound Summary page

**Fig. 4:**
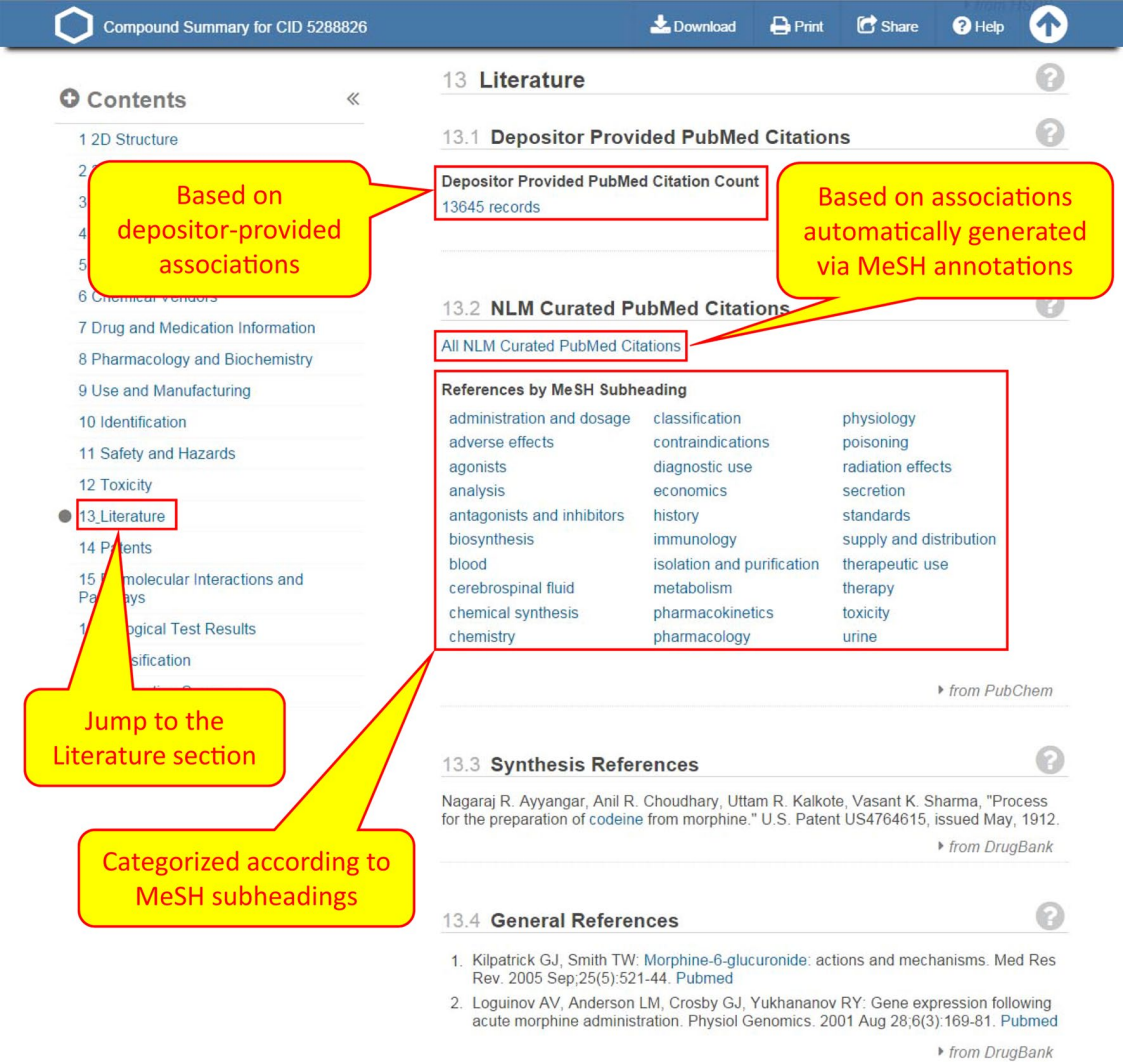
The literature section of the Compound Summary page (DocSum) for CID 5288826 (morphine). Clicking the “Literature” section in the Table of Contents allows users to jump to the literature section, which consists of two subsections: depositor-provided and NLM-curated PubMed citations

The Compound Summary page of a compound provides an aggregated view of all available information on that compound collected from various data sources (Fig. 4). When a compound has literature information, this will be shown in the Literature section. A link to PubMed articles associated with the compound via depositor-provided cross-references is displayed under the Depositor Provided PubMed Citations section, and a link to those associated with the compound via common MeSH annotations is shown under the NLM Curated PubMed Citations section. In some cases, the per compound PMID associations derived via MeSH annotations are categorized according to MeSH subheadings, which allow for describing particular aspects of a subject. MeSH terms associated with a compound are displayed under the Classification section.

### Entrez filters from PubChem records to PubMed articles

**Fig. 5:**
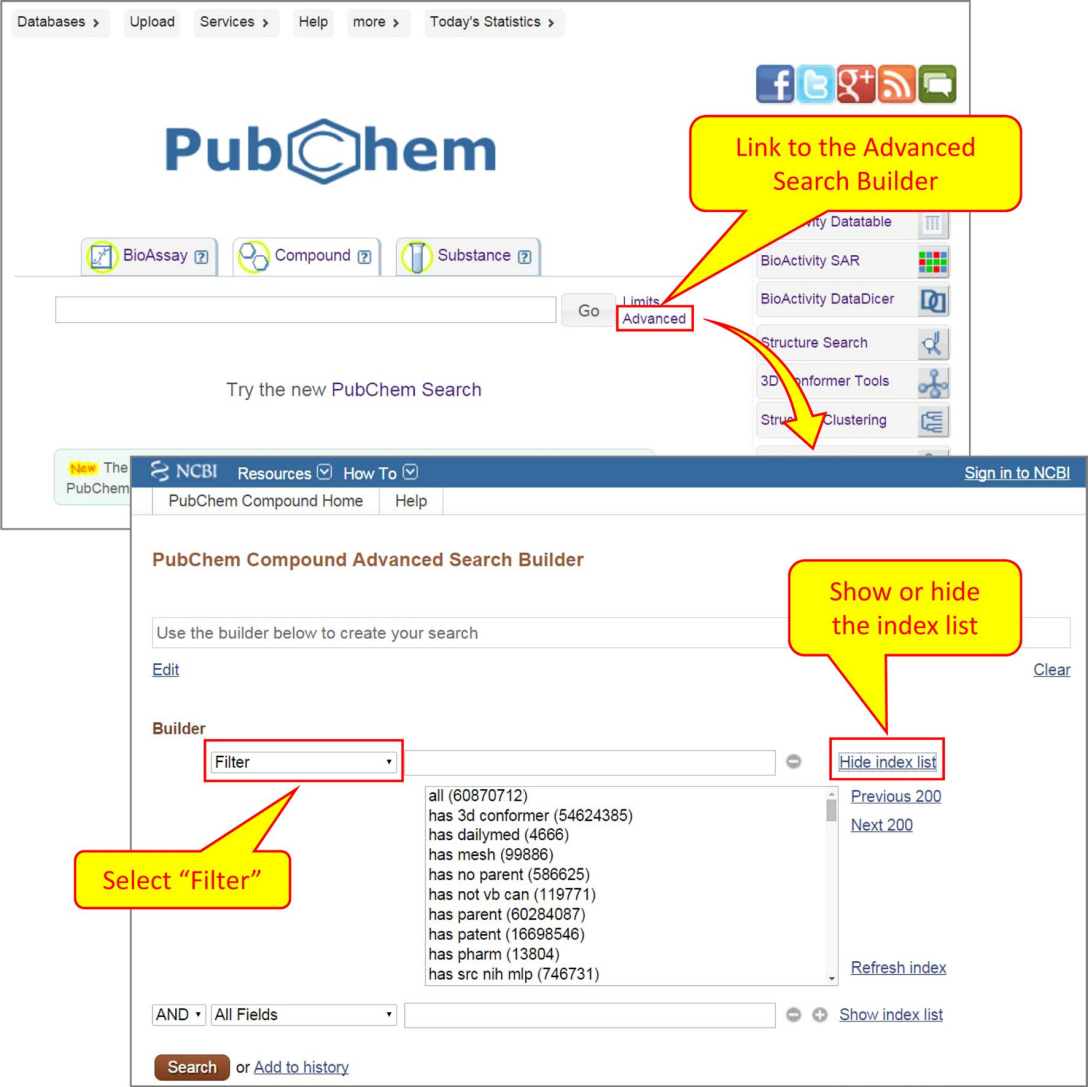
Retrieving compound records with a particular property using the Entrez filters. Selecting the “Filter” option under the drop-down menu on the PubChem Compound Advanced Search Builder allows you to retrieve compounds with a particular property or annotation. Available filters can be shown or hidden by clicking the “Show/Hide index list” button

One may want to retrieve all PubChem records associated with PubMed articles. This can be done using Entrez filters, which indicate whether or not a given record in an Entrez database has a particular type of annotations. The Entrez filters may be used to subset other Entrez searches according to this annotation type, by adding the filter to the query string. Importantly, Entrez filters are closely related to Entrez links in that many Entrez filters are generated by checking whether a given record in an Entrez database has an Entrez link to a record in the same or different Entrez database. The filters for each Entrez database may be listed by going to the advanced search page of each database, selecting “Filter” from the “All Fields” dropdown, and clicking “Show index list” (Fig. 5).

Table 4 lists the Entrez Filters available in PubChem that can be used to get the list of records associated with PubMed articles. As mentioned in the previous section, a DocSum page for a compound search result (Fig. 3) shows three different Entrez links between compounds and PubMed articles. These three links are used to generate three Entrez filters that indicate the presence of associated PubMed articles for compound records: (1) pccompound_pubmed, (2) pccompound_pubmed_mesh, and (3) pccompound_pccompound_publisher. The “pccompound_pubmed” filter allows one to retrieve compound records with cross-references provided *toPubChem by data contributors*. The “pccompound_pubmed_publisher” filter retrieves those with cross-references to PubMed articles that are provided *toPubMed by publishers*. The “pccompound_pubmed_mesh” filter retrieves those with computationally generated links to PubMed articles that have a common MeSH annotation, as explained in the previous section.

**Table 4:** Entrez filters used to search for records with MeSH annotations

Entrez filter	Description
Compound database	
has_mesh	Equivalent to “pccompound_mesh”
has_pharm	Equivalent to “pccompound_mesh_pharm”
pccompound_mesh	Select compounds annotated with MeSH terms. Equivalent to “has_mesh”
pccompound_mesh_pharm	Select compounds annotated with MeSH Pharmacological Actions. Equivalent to “has_pharm”
pccompound_pmc	Select compounds that have associated full-text articles in PubMed Central
pccompound_pubmed	Select compounds that have depositor-provided cross-references to PubMed articles
pccompound_pubmed_mesh	Select compounds associated with PubMed abstracts that are annotated with common MeSH annotations
pccompound_pubmed_publisher	Select compounds that have cross-references to PubMed articles, provided to PubMed by publishers
BioAssay database	
pcassy_pmc	Select assays that have associated full-text articles in PubMed Central
pcassay_pubmed	Select assays that have depositor-provided cross-references to PubMed articles
pcassay_pubmed_major	Select assays that have cross-references to the PubMed articles that contains the original bioactivity data in the assays

While the “pccompound_mesh” filter retrieves any compounds with MeSH annotations, the “pccompound_mesh_pharm” filter selects those with Pharmacological Action annotations. These filters are equivalent to the “has_mesh” and “has_pharm” filters, respectively. The “pccompound_pmc” filter allows one to choose compounds that have associated full-text articles in PubMed Central.

The Substance database also has Entrez filters that are similar to those for compound records listed in Table 4. For example, the “pcsubstance_pubmed” and “pcsubstance_pubmed_publisher” filters can be used to find substance records with cross-references to PubMed that are provided by PubChem depositors to PubChem and by publishers to PubMed, respectively.

### Programmatic access to literature information in PubChem

Literature information in PubChem can be programmatically accessed through E-Utilities [30] or PUG-REST [31]. More detailed information on programmatic access to PubChem is given elsewhere [31].

## Discussion

**Fig. 6:**
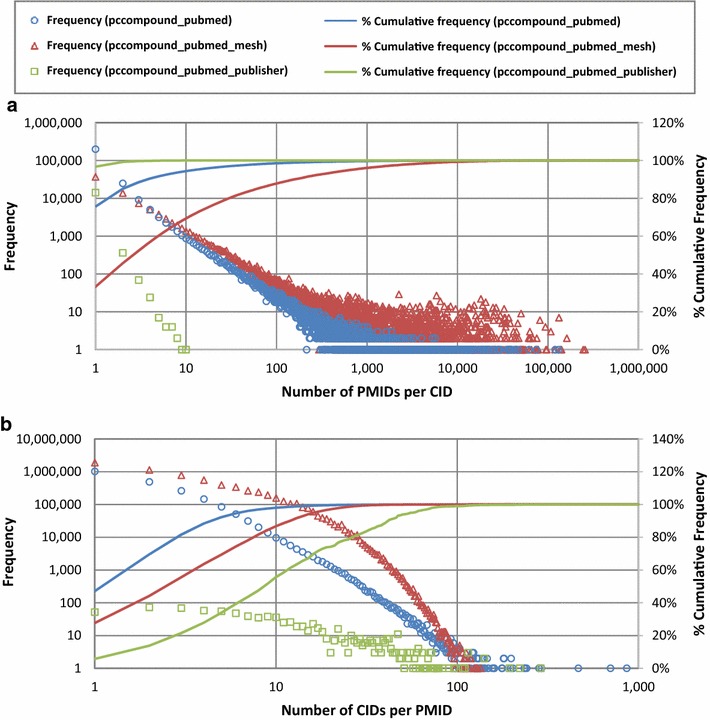
Distribution of PMIDs per CID and CIDs per PMID for three types of Entrez links. Distributions of **a** PMIDs per CID and **b** CIDs per PMID are shown for three Entrez links between PubChem Compound and PubMed: “pccompound_pubmed”, “pccompound_pubmed_mesh”, and “pccompound_pubmed_publisher”. See texts for the description of these links

Figure 6 shows the frequencies of PMIDs per CID and CIDs per PMID for the three types of CID-PMID associations: those provided to PubChem by PubChem depositors (pccompound_pubmed), those derived by PubChem through matching between chemical names and MeSH terms (pccompound_pubmed_mesh), and those provided to PubMed by journal publishers (pccompound_pubmed_publisher). Note that the frequencies for the pccompound_pubmed_publisher links are much lower than the other two link types because only a small number of publishers submit CID-PMID associations to PubMed. Therefore, this section focuses the other two link types.

The majority of compounds have no more than ten associated PMIDs (i.e., 95 % of CIDs with depositor-provided PMIDs and 70 % of CIDs with automated annotations via MeSH). However, some compounds are associated with many PMIDs. For example, d-glucose has as many as 130,545 depositor-provided PMIDs and 132,017 PMIDs generated via MeSH.

**Fig. 7:**
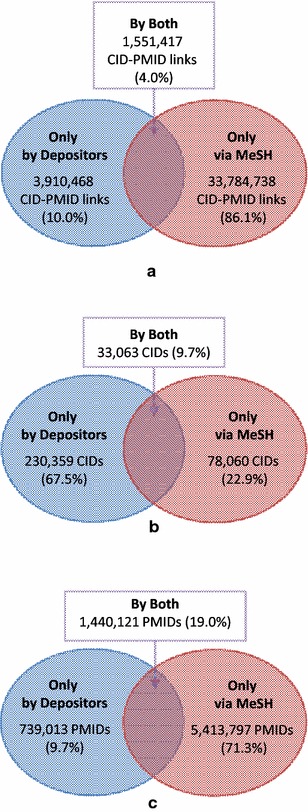
Venn diagrams for depositor-provided CID-PMID associations and those generated via MeSH. The Venn diagrams compare depositor-provided CID-PMID associations and automated annotations via MeSH in terms of **a** the number of CID-PMID associations, and **b** CIDs and **c** PMIDs involved in these associations

The Venn diagrams shown in Fig. 7 illustrate the overlap between CID-PMID associations provided by depositors and derived annotations via MeSH. Among the 39.2 million CID-PMID associations, only 4 % overlap, being both depositor-provided and NLM-curated, indicating that the two types of associations are nearly orthogonal and complement each other. Of these 4 % overlapping CID-PMID pairs, the CIDs and PMIDs that make them up correspond to 10 and 19 % of all CIDs and all PMIDs found in all CID-PMID pairs, respectively. Importantly, although depositor-provided CID-PMID links are much fewer in number than those generated via MeSH, there are more CIDs involved in the depositor-provided links than in those derived via MeSH.

It is worth noting that the CID-PMID associations via MeSH tend to be limited to chemicals that are sufficiently well known to be included in MeSH. These chemicals correspond to only a small fraction of all chemicals studied in scientific articles contained in PubMed. Therefore, associations generated via MeSH may ignore specific chemicals (favoring instead a chemical class) unless they are already included in MeSH or sufficiently noteworthy to be specifically added. With that said, depositor-provided CID-PMID associations do not suffer the same limitation as MeSH and provide a greatly increased number of chemicals mentioned in the biomedical literature, as suggested in Fig. 7.

However, a caveat against depositor-provided associations is that the accuracy and usefulness of these links solely rely on individual depositors’ quality control efforts, as PubChem does not (currently) provide an independent quality control mechanism. To address this, PubChem is exploring a few possibilities. For example, one option is to process PubMed abstracts (and PubMed Central full-text papers) using text-mining software for chemical recognition (in text, tables, and figures). The resulting CID-PMID associations can be used to cross-validate the existing depositor-provided associations and to better understand the context of the chemical mention. Annotation of CID-PMID associations (e.g., location, context) may help prioritize the display of PMIDs associated to a given compound.

## Conclusions

Literature information available in PubChem for substances, compounds and assays, as well as how this information can be accessed, was described. Individual data contributors provide PubChem with cross-references between chemical substances and PubMed articles that contain information on that substance. From these SID-PMID cross-references, PubChem generates cross-references between the corresponding compound and the PubMed articles (i.e., CID-PMID cross-references). Data contributors can also supply a list of PMIDs for scientific articles that have information relevant to a given assay record. These articles may contain various kinds of information related to the assay, including experimental protocols, assay targets, diseases associated with the targets, and known ligands that bind the targets. Of particular interest, some data contributors provide bioactivity data extracted from literature through manual curation or data mining and are an important source of bioactivity information in PubChem that complement HTS data from the now-concluded NIH Molecular Libraries Program and other HTS projects. In addition to community-provided literature information, PubChem generates Entrez links between PubChem records and PubMed articles that share the same MeSH annotation. This automated process allows PubChem users to leverage the biomedical literature and its MeSH indexing for search and analysis purposes.

Some journals, such as *Nature Chemical Biology*, provide PubChem with information on chemicals that appear in their newly published articles. This enables PubChem to direct users to the new articles on the journal web site, even before their abstracts become available in PubMed. In turn, the publisher can provide their readers with access to comprehensive information available in PubChem about the chemicals mentioned in a given article. This exemplifies the mutual benefit of concurrent publication of scientific articles in journals and associated data in public databases.

Literature information, both provided by depositors and derived via MeSH, can be accessed from the DocSum page of an Entrez search result, or from the Compound Summary, Substance Record, or BioAssay Record page. Users can also retrieve PubChem records associated with scientific articles, using appropriate Entrez filters. These tools allow PubChem users to more readily explore information available in literature related to PubChem records.
